# Structured RNA Contaminants in Bacterial Ribo-Seq

**DOI:** 10.1128/mSphere.00855-20

**Published:** 2020-10-21

**Authors:** Brayon J. Fremin, Ami S. Bhatt

**Affiliations:** a Department of Genetics, Stanford University, Stanford, California, USA; b Department of Medicine (Hematology), Stanford University, Stanford, California, USA; University of British Columbia

**Keywords:** RNA structure, metagenomics, metatranscriptomics, microbiome

## Abstract

Ribosome profiling (Ribo-Seq) is a powerful method to study translation in bacteria. However, Ribo-Seq signal can be observed across RNAs that one would not expect to be bound by ribosomes. For example, Escherichia coli Ribo-Seq libraries also capture reads from most noncoding RNAs (ncRNAs). While some of these ncRNAs may overlap coding regions, this alone does not explain the majority of observed signal across ncRNAs. These fragments of ncRNAs in Ribo-Seq data pass all size selection steps of the Ribo-Seq protocol and survive hours of micrococcal nuclease (MNase) treatment. In this work, we specifically focus on Ribo-Seq signal across ncRNAs and provide evidence to suggest that RNA structure, as opposed to ribosome binding, protects them from degradation and allows them to persist in the Ribo-Seq sequencing library preparation. By inspecting these “contaminant reads” in bacterial Ribo-Seq, we show that data previously disregarded in bacterial Ribo-Seq experiments may, in fact, be used to gain partial information regarding the *in vivo* secondary structure of ncRNAs.

**IMPORTANCE** Structured ncRNAs are pivotal mediators of bioregulation in bacteria, and their functions are often reliant on their specific structures. Here, we first inspect Ribo-Seq reads across noncoding regions, identifying contaminant reads in these libraries. We observe that contaminant reads in bacterial Ribo-Seq experiments that are often disregarded, in fact, strongly overlap with structured regions of ncRNAs. We then perform several bioinformatic analyses to determine why these contaminant reads may persist in Ribo-Seq libraries. Finally, we highlight some structured RNA contaminants in Ribo-Seq and support the hypothesis that structures in the RNA protect them from MNase digestion. We conclude that researchers should be cautious when interpreting Ribo-Seq signal as coding without considering signal distribution. These findings also may enable us to partially resolve RNA structures, identify novel structured RNAs, and elucidate RNA structure-function relationships in bacteria at a large scale and *in vivo* through the reanalysis of existing Ribo-Seq data sets.

## Abstract (precis)

Structured ncRNAs are pivotal mediators of bioregulation in bacteria, and their functions are often reliant on their specific structures. Here, we first inspect Ribo-Seq reads across noncoding regions, identifying contaminant reads in these libraries. We observe that contaminant reads in bacterial Ribo-Seq experiments that are often disregarded, in fact, strongly overlap with structured regions of ncRNAs. We then perform several bioinformatic analyses to determine why these contaminant reads may persist in Ribo-Seq libraries. Finally, we highlight some structured RNA contaminants in Ribo-Seq and support the hypothesis that structures in the RNA protect them from MNase digestion. We conclude that researchers should be cautious when interpreting Ribo-Seq signal as coding without considering signal distribution. These findings also may enable us to partially resolve RNA structures, identify novel structured RNAs, and elucidate RNA structure-function relationships in bacteria at a large scale and *in vivo* through the reanalysis of existing Ribo-Seq data sets.

## OBSERVATION

Ribosome profiling (Ribo-Seq) in bacteria is a method that enriches for ribosome-protected RNAs and therefore, enables the study of active translation events (1, 2). Ribo-Seq protocols enrich for monosomes using sucrose density gradients (1) or size exclusion columns (3) but do not specifically isolate monsomes. Ribo-Seq is especially challenging in bacteria because, unlike in yeast and other eukaryotes, bacteria have a broad size distribution of ribosome-protected footprints, ranging from 15 to 40 nucleotides (4). The size range that should be selected can vary across Ribo-Seq protocols; at present, most published Ribo-Seq experiments on bacteria have targeted a size range of 15 to 45 nucleotides, as was used by Li et al. (1). Hence, compared to eukaryotic ribosome profiling protocols, bacterial ribosome profiling protocols must adopt less stringent size selection to comprehensively capture biologically relevant, actively translated RNAs.

Imperfect monosome isolation and selection of a wider range of fragments would enable RNA contaminants of diverse sizes to persist in bacterial Ribo-Seq libraries, including structured noncoding RNAs (ncRNAs) (5). These structured noncoding contaminants have been acknowledged in the literature (5), but they have not been thoroughly investigated and are often overlooked when analyzing Ribo-Seq results (6). We hypothesize that some of these contaminants survive MNase treatment because they are protected from degradation by virtue of their secondary structure. This hypothesis is conceptually similar to one utilized in the method FragSeq (7); however, FragSeq utilizes a different enzyme, nuclease P1, for fragmentation and aims to probe specific secondary structures of RNA via fragmentation patterns *in vitro* (7). Here, we propose that instead of disregarding these contaminant signals in Ribo-Seq libraries, the micrococcal nuclease (MNase) treatment, much like nuclease P1 in FragSeq (7), may provide valuable insight in identifying RNA structures *in vivo*.

To test the hypothesis that structured ncRNAs persist in Ribo-Seq libraries, we analyzed existing E. coli Ribo-Seq data sets to determine whether these ncRNAs were detected. We quantified Ribo-Seq and transcriptome sequencing (RNA-Seq) reads across 65 known ncRNAs in E. coli MG1655 (see Table S1 in the supplemental material). All of these ncRNAs were found to be transcribed (reads per kilobase million [RPKM] > 10) in RNA-Seq data from Li et al. (1). Of the 65 known ncRNAs, 61 (94%) produced a Ribo-Seq signal (RPKM > 10) in Ribo-Seq experiments from Li et al. (1) and in Ribo-Seq of MG1655 E. coli performed in our laboratory and recently reported (8) (Table S1). Widespread coding by bacterial small RNAs has been described (9, 10). When we performed an open reading frame (ORF) calling experiment on E. coli, we found that 43 of the 65 ncRNAs did not overlap with an ORF with coding potential or a ribosome binding site, and 2 of the expressed ncRNAs did not overlap any possible ORF. This suggests that the signal cannot be explained by overlapping ORFs.

10.1128/mSphere.00855-20.2 TABLE S1 Comprehensive analysis of ncRNAs in E. coli across Ribo-Seq data sets quantifying Ribo-Seq signal and potential for ORFs. We provide RPKM calculations for Ribo-Seq performed on E. coli (MG1655) in-house (8), MetaRibo-Seq performed on a fecal E. coli strain (8), and Ribo-Seq and RNA-Seq performed on E. coli (MG1655) by Li et al. in 2014 (1). Additionally, we predicted all possible ORFs and denoted which ncRNAs overlapped with potential ORFs. We also break down these overlapping ORFs to include those that have either a positive start site or coding potential assessed via Prodigal (18) or were small (less than 50 amino acids). Download Table S1, XLSX file, 0.01 MB. Copyright © 2020 Fremin and Bhatt. 2020 Fremin and Bhatt This content is distributed under the terms of the Creative Commons Attribution 4.0 International license.

**FIG 1:**
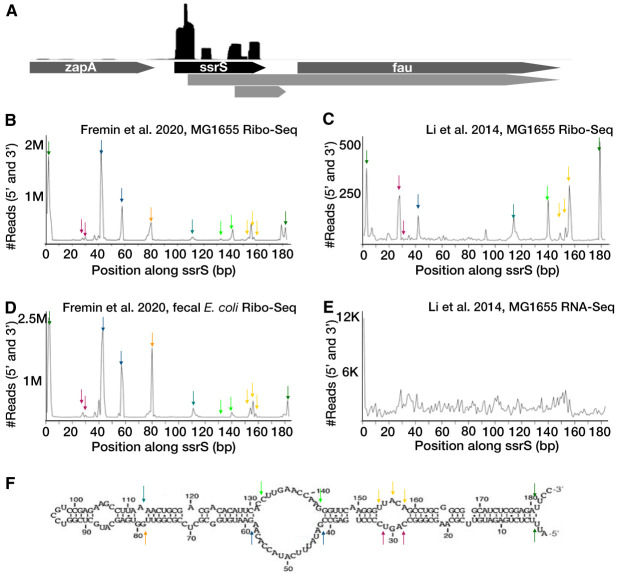
Ribo-Seq fragmentation patterns of *ssrS* suggest that RNA secondary structures protect it from MNase. (A) Interactive Genome Browser (IGV) view of Ribo-Seq signal across *ssrS*. The black trace above the displayed genomic regions represents the relative coverage of each region by individual sequencing reads. The genes are shown in dark gray. Possible ORFs are shown in light gray. (B) Quantification of the 3′ and 5′ ends of Fremin et al. 2020 (8) Ribo-Seq reads mapping to *ssrS* in E. coli MG1655. Arrows indicate peaks in signal. (C) Quantification of the 3′ and 5′ ends of Li et al. 2014 (1) Ribo-Seq reads mapping to *ssrS* in E. coli MG1655. (D) Quantification of the 3′ and 5′ ends of Fremin et al. 2020 (8) MetaRibo-Seq reads mapping to *ssrS* in E. coli within a fecal sample. (E) Quantification of the 3′ and 5′ ends of Li et al. 2014 (1) RNA-Seq reads mapping to *ssrS* in E. coli MG1655. (F) Characterized structure of *ssrS* in E. coli. This structure diagram was created using data from previous work (11–13). Arrows indicate relative positions comparing line graphs (A to D) to this structure diagram.

To test whether fragmentation seen in Ribo-Seq libraries correlates with the structural accessibility of RNAs, we visualized the fragmentation pattern across a highly transcribed structured RNA, *ssrS*, native to E. coli (Fig. 1). The structure of *ssrS* in E. coli has been previously validated (11–13). First, we found that Ribo-Seq reads were specific to the boundaries of *ssrS* (Fig. 1A). Even if we were to give the “benefit of the doubt” that the two possible ORFs overlapping *ssrS* were in fact coding, it still would not explain all of the reads that specifically align within the *ssrS* boundaries that do not overlap potential ORFs. This suggests that the Ribo-Seq signal observed is a contaminating noncoding signal. When viewing Ribo-Seq signal for other structures, *ffn*, *sokC*, *sokX*, and *spf* (see Fig. S1 in the supplemental material), we also find that contamination best explains the signal. Focusing only on the 5′ and 3′ ends of reads, representing where MNase fragmentation of the RNA occurred, we find that the ends of Ribo-Seq reads were overrepresented specifically at junctions between structured and unstructured regions of *ssrS*. This association was reproducibly observed across studies—in our Ribo-Seq experiments on E. coli MG1655 (Fig. 1B to D), similar experiments performed by Li et al. (1), and from MetaRibo-Seq experiments carried out on a fecal sample containing a clinical E. coli strain, referred to in a previous manuscript as sample E (8). Importantly, this fragmentation pattern was not reproduced in RNA-Seq libraries that were not exposed to MNase digestion (1) (Fig. 1E). Therefore, it is likely that *in vivo* secondary structures within *ssrS* protect it from MNase digestion in Ribo-Seq protocols. These fragments are then retained after monosome recovery and fragment size selection.

10.1128/mSphere.00855-20.1 FIG S1 IGV visualizations of Ribo-Seq reads overlapping ncRNAs in E. coli. (A to D) We show Ribo-Seq signal in E. coli from Li et al. (1) across ncRNAs, specifically *ffs*, *sokX*, *sokC*, and *spf*. These are examples of ncRNAs with Ribo-Seq reads that also overlap possible ORFs. Download FIG S1, PDF file, 0.7 MB. Copyright © 2020 Fremin and Bhatt. 2020 Fremin and Bhatt This content is distributed under the terms of the Creative Commons Attribution 4.0 International license.

**FIG 2:**
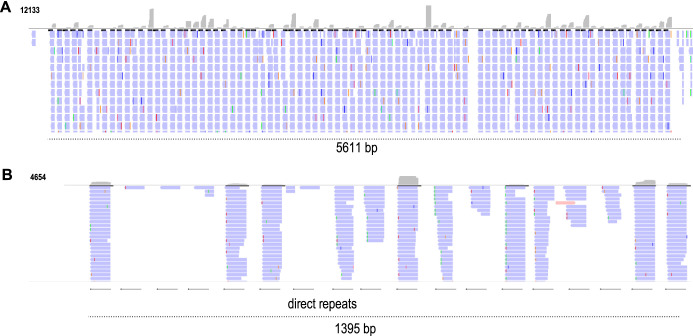
MetaRibo-Seq signal across CRISPR arrays in two gut commensals suggests that secondary structures of direct repeats protect it from MNase. (A) Ribo-Seq signal across a CRISPR array containing 84 repeats, predicted by minCED (25). This is found in *Ruminococcus* sp. strain UNK.MGS-30. For reference, this was predicted from sample C in previous work (8). (B) Ribo-Seq signal across an 18-repeat CRISPR array in Ruminococcus lactaris, also predicted by minCED (25). For reference, this was predicted from sample A in previous work (8). Arrows indicate direct repeats.

To further test the hypothesis that these contaminant fragments of RNA persist due to their secondary structure, we next turned our attention to CRISPR arrays from *Ruminococcus.* We hypothesized that since direct repeats are the only structured regions of RNA in CRISPR arrays, only these would survive MNase treatment and therefore be represented in Ribo-Seq data. To test this, we inspected MetaRibo-Seq signal distribution along CRISPR arrays and found a strong enrichment for structured repeats in the CRISPR arrays (Fig. 2). For example, a CRISPR array containing 18 repeats in Ruminococcus lactaris, a human gut commensal, contained Ribo-Seq signal specific to each of the 18 repeats in the array (Fig. 2B). This suggested that MNase was able to digest spacer regions in these CRISPR arrays but was unable to digest the structured direct repeat regions. Notably, this reinforces our hypothesis that structured regions of ncRNAs escape MNase digestion and therefore are represented in Ribo-Seq experiments.

While this approach represents an exciting new repurposing of existing Ribo-Seq data, there are several limitations to using contaminant Ribo-Seq signals to gain insights into the structure of RNAs. First, this method is not designed to study structured RNAs and in fact contains steps to actively filter out such contaminants. Ribo-Seq protocols enrich for ribosomes and restrict RNA sequences to a specific size range—therefore, many fragments of RNA that are of structural interest are experimentally removed. Further, this process of eliminating RNA fragments results in a fragmentation profile that is incomplete. Additionally, we cannot assume that all contaminant fragments are retained after monosome recovery. The absence of a peak in a Ribo-Seq fragmentation profile for a given structured RNA does not imply that the specific structure is not there. We refrain from drawing conclusions from the intensity of any given peak as this could be influenced by transcript abundance, MNase specificity, and fragment length. Methods like FragSeq (7) and Shape-Seq (14, 15) will undoubtedly be more sensitive and provide a more comprehensive catalog of structured RNAs. Additionally, MNase may not be the best enzyme for such fragmentation. From a methodological standpoint, Ribo-Seq cannot match the resolution or completeness of existing technologies to probe for the structures of RNAs. That said, the concept that ncRNA retained in Ribo-Seq protocols have RNA structure appears to be a supportable hypothesis as to how these fragments persist after MNase treatment. Alternatively, it is also possible that contaminant fragments are created when other proteins, not ribosomes, protect regions from MNase digestion. As Ribo-Seq protocols continue to improve, the existence of these contaminants may also diminish.

Despite these limitations, there are several notable strengths to these findings. First, it allows us to better understand the limitations of Ribo-Seq. Second, it provides an explanation as to why these contaminants exist in the data. Third, it allows us to find utility in these contaminant Ribo-Seq signals to gain insight into structured RNAs. Currently, there is a plethora of Ribo-Seq data, especially with the development of MetaRibo-Seq and the ability to capture the ribosome profile of thousands of taxa at once. To our knowledge, no one has performed a method like FragSeq (7) or Shape-Seq (14) on a complex fecal community. Ribo-Seq has the potential advantage of partially capturing *in vivo* RNA structures, in high throughput, and can immediately be applied to the vast existing data sets. Additionally, Ribo-Seq data may be leveraged to identify novel structured RNAs, many of which are yet to be discovered (16).

In summary, here we highlight contaminant Ribo-Seq signals and propose an explanation for why these fragments exist in the data. First, we find that most ncRNAs in E. coli contain Ribo-Seq signal that cannot be entirely explained by coding regions. Second, we analyzed the fragmentation pattern of a well-established structured RNA, *ssrS*, in E. coli. We observed that the ends of Ribo-Seq reads accumulated at junctions between structured and unstructured regions of the *ssrS* RNA, suggesting that the RNA structure is protected against MNase digestion, akin to FragSeq (7). Third, we inspected the signal distribution along CRISPR arrays in Ruminococcus lactaris. We observed that structured repeats within CRISPR arrays (16) retained Ribo-Seq reads while spacer regions did not retain reads, suggesting that the structure of the direct repeats was protected from MNase. By focusing on these contaminants in Ribo-Seq data, we specifically addressed their prevalence, why they exist in this data type, and how they may be useful to researchers interested in the *in vivo* structure of RNAs.

### Methods.

**(i) Data download.** Reads from all samples used are publicly available. The in-house-generated data can be found under BioProject accession no. PRJNA510123 (8, 17). Ribo-Seq and RNA-Seq for E. coli generated by Li et al. in 2014 can be found under BioProject accession no. PRJNA232843 (1).

**(ii) Genome annotation.** To annotate all possible genes in E. coli, we used Prodigal (18) with a lower length cutoff of 15 nucleotides to capture small ORFs also (19). We used the -s parameter with Prodigal to access the intermediate output, which assigned start site scores and coding potential scores to every possible ORF. CRISPR arrays were predicted from reference genomes using minCED (25) as a part of Prokka v1.12 (20).

**(iii) Read mapping.** Reads were trimmed with trim galore version 0.4.0 using cutadapt 1.8.1 (21) with flags –q 30 and –illumina. Reads were mapped to the annotated assemblies using bowtie version 1.1.1 (22). Reads were counted using bedtools (23) multicov. The 5′ and 3′ positions of reads were determined using bedtools (23) genomecov. When analyzing fragmentation patterns of reads, reads derived from fragments longer than the read length were removed from the analysis. Interactive Genome Browser (IGV) (24) was used to visualize coverage. Reads per kilobase million (RPKM) calculations were performed using in-house scripts.
