# Supplementary figures and images for: Structured RNA Contaminants in Bacterial Ribo-Seq
Source: mSphere. 2020 Oct 21;5(5):e00855-20. doi: 10.1128/mSphere.00855-20 (PMC7580957; doi:10.1128/mSphere.00855-20)

**A**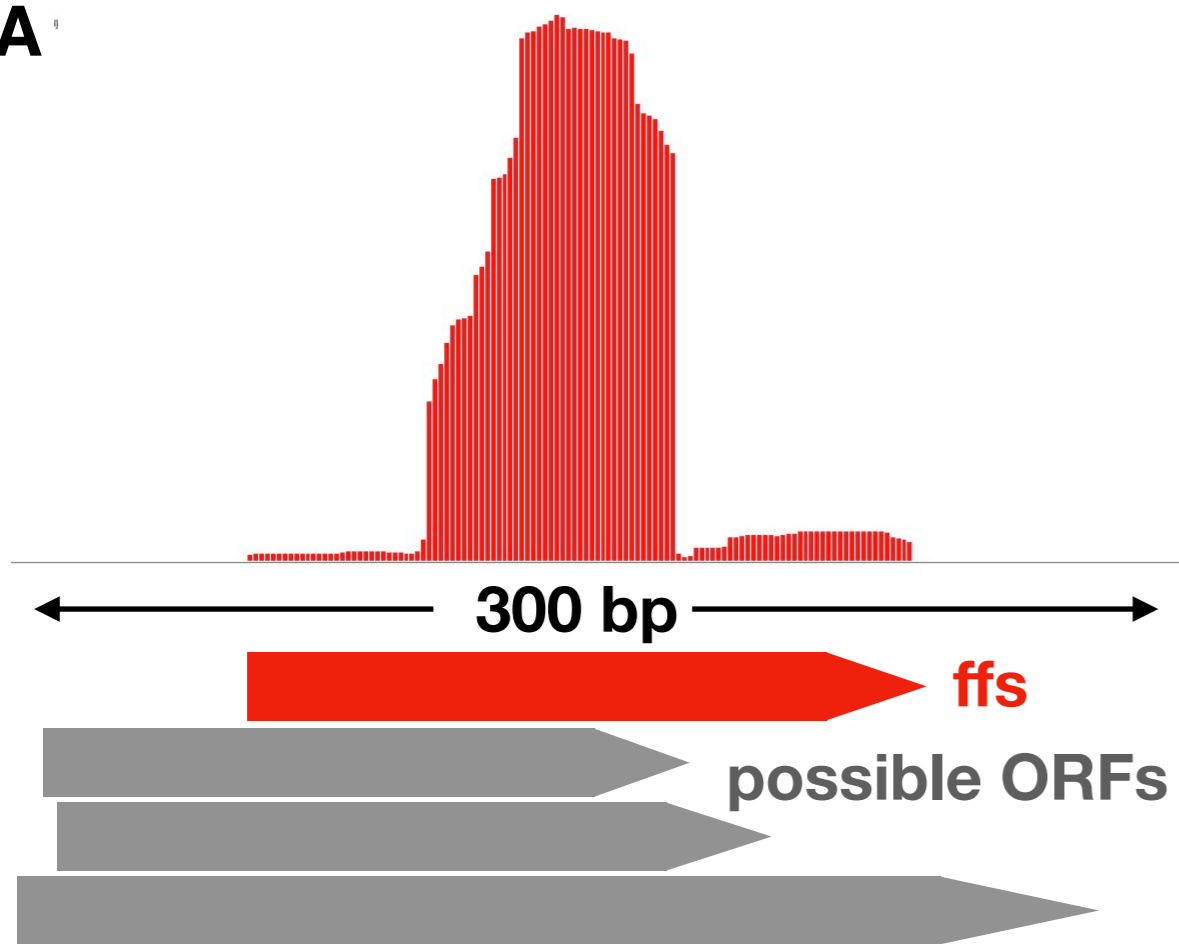**B**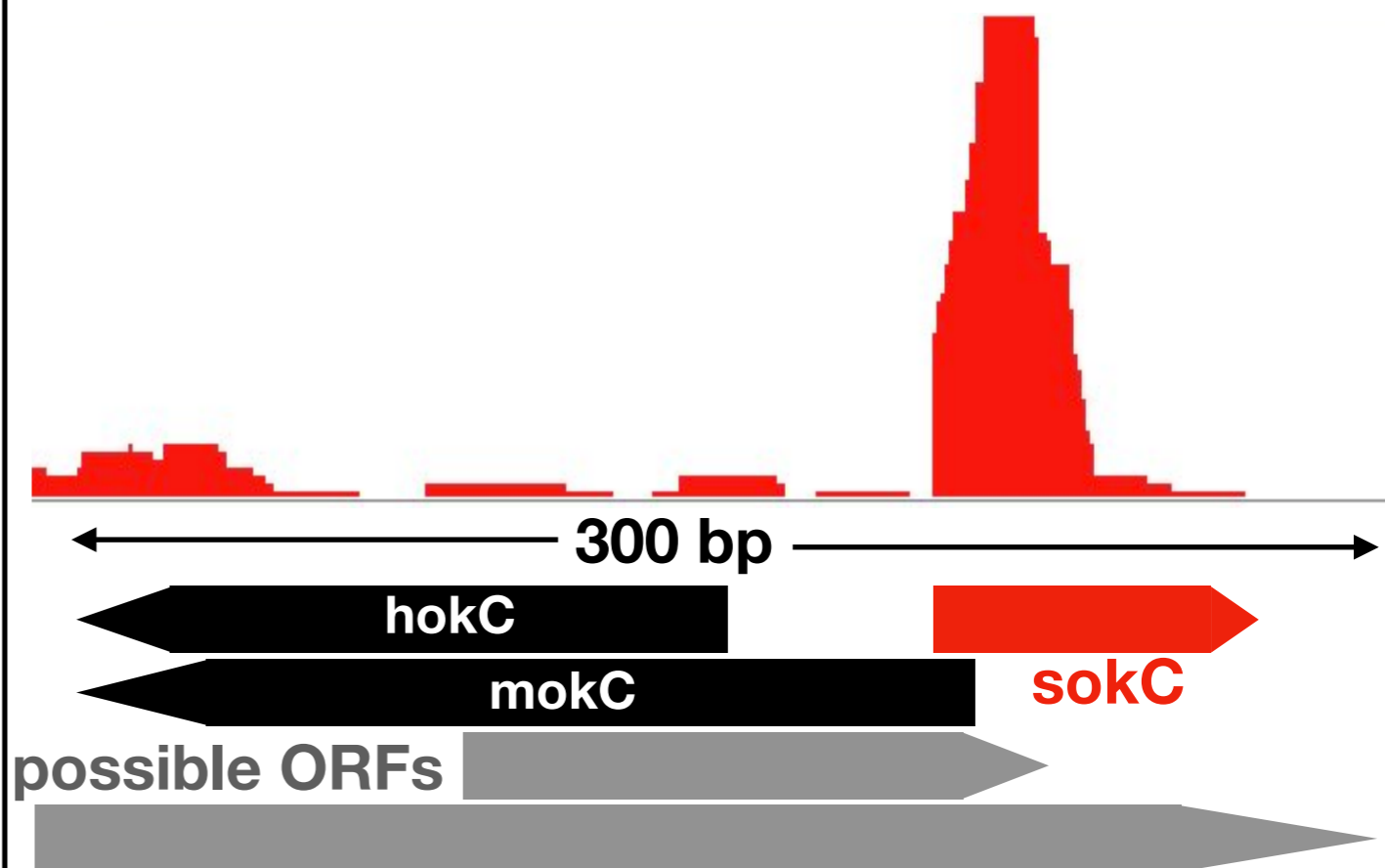**C**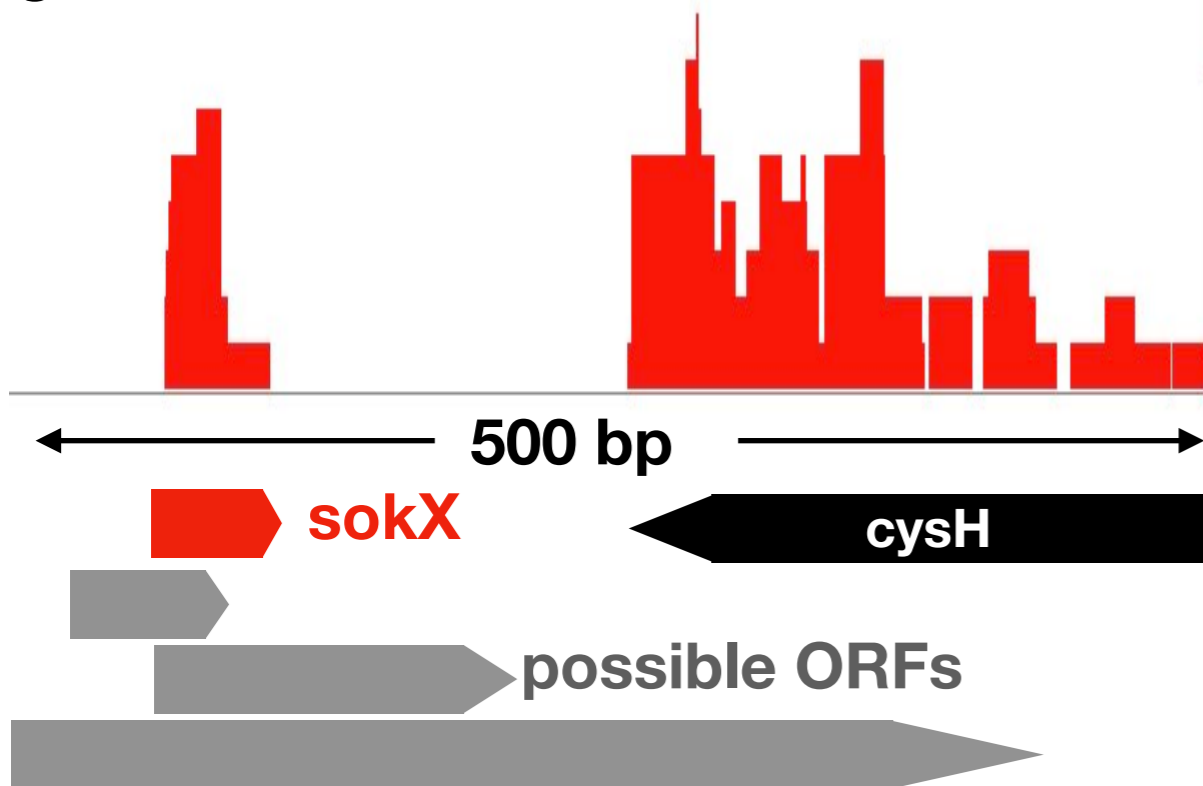**D**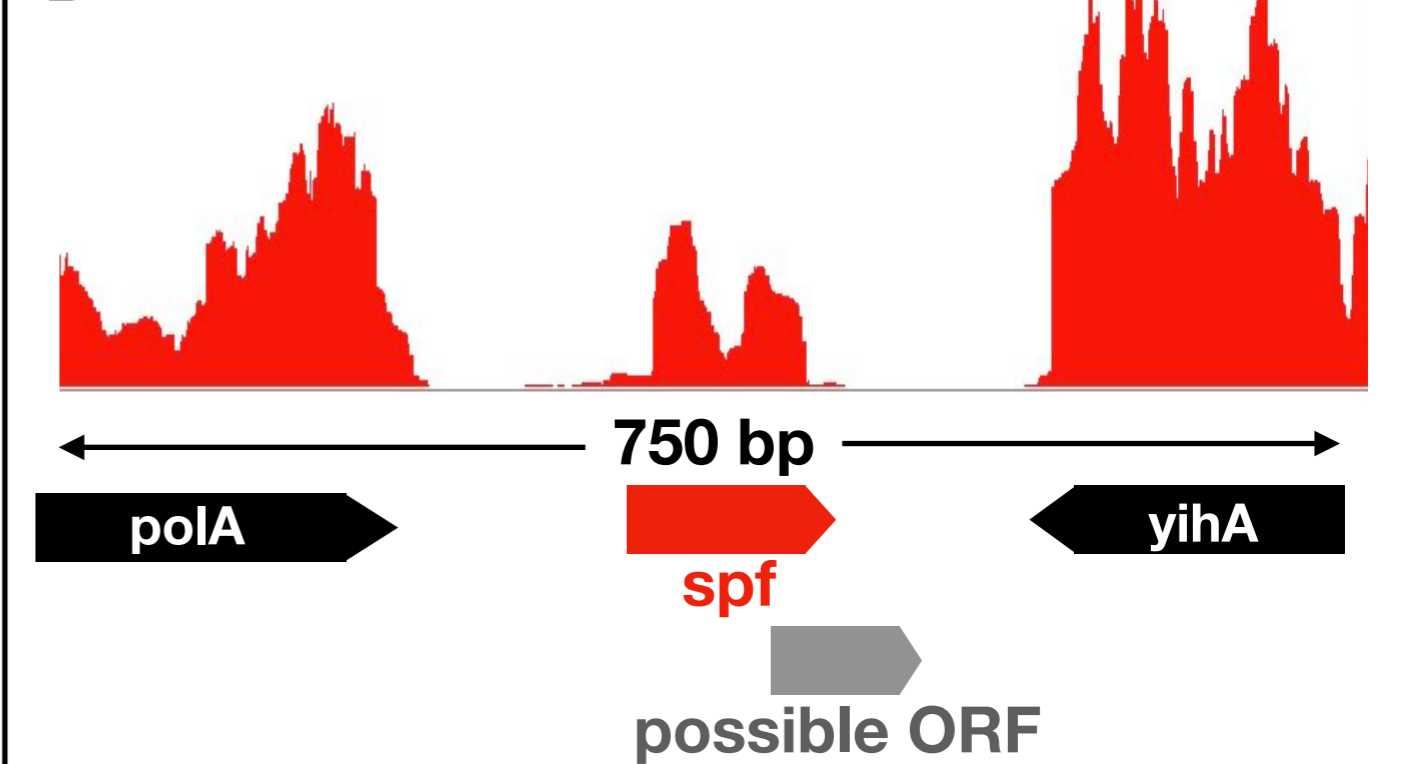

Supplement: FIG S1 [file mSphere.00855-20-sf001.pdf]
